# Expanding prenatal diagnosis: clinical utility of prenatal whole-exome sequencing in a Romanian case series with fetal anomalies

**DOI:** 10.25122/jml-2026-0116

**Published:** 2026-07

**Authors:** Ileana-Delia Manea-Săbău, Adina-Elena Nenciu, Valentin Nicolae Varlas, Antoanela Curici, Maria Riza, Mihai Mitroi, Raluca Mihaela Radu, Viorel Suciu-Lazar, Iuliana Ceaușu

**Affiliations:** 1 Doctoral School, Carol Davila University of Medicine and Pharmacy, Bucharest, Romania; 2 Synevo Romania, Bucharest, Romania; 3 Department of Medical Genetics, Carol Davila University of Medicine and Pharmacy, Bucharest, Romania; 4 Department of Obstetrics and Gynecology I, Dr. I. Cantacuzino Hospital, Carol Davila University of Medicine and Pharmacy, Bucharest, Romania; 5 Carol Davila University of Medicine and Pharmacy, Bucharest, Romania; 6 Department of Cellular and Molecular Biology and Histology, Carol Davila University of Medicine and Pharmacy, Bucharest, Romania; 7 Alessandrescu Rusescu National Institute for Mother and Child Health, Bucharest, Romania; 8 Pelican Hospital, Oradea, Romania

**Keywords:** prenatal diagnostics, array CGH, exome sequencing, next generation sequencing, Romanian population, amniotic fluid

## Abstract

The prenatal diagnostic field is rapidly expanding internationally, creating a growing need for guidelines and for a better understanding of the clinical utility of new tests across different clinical settings. This study aimed to investigate the diagnostic yield of prenatal whole exome sequencing (WES) and to assess its clinical utility and impact on outcomes among families with high-risk pregnancies and the multidisciplinary teams supervising the cases. We retrospectively reviewed indications, fetal phenotypes, results, turnaround time, and the clinical impact of WES in 10 pregnancies from the Romanian population, investigated in a private clinical diagnostic laboratory between January 2024 and March 2026. The diagnostic yield of prenatal WES was 50% in this case series of Romanian pregnancies with ultrasound-detected malformations and a previous negative microarray result. Seven out of ten patients opted for medical termination of pregnancy. Our findings are consistent with those of other national and international studies, while highlighting the need for improved clinical pathways and guidelines to support appropriate test selection and prenatal management. The multidisciplinary team considered prenatal WES an efficient tool for prenatal diagnosis, with potential to inform pregnancy management, including the choice of maternity hospital level, delivery planning, and personalized neonatal care.

## Introduction

Prenatal diagnostics encompasses a range of tests that use advanced cytogenetic and molecular technologies to evaluate fetal health *in utero*. Current methods have different indications and can diagnose multiple fetal genetic disorders. These approaches range from classical techniques such as conventional karyotyping, fluorescence in situ hybridization (FISH), and molecular karyotyping to next-generation sequencing panels and comprehensive prenatal tests such as whole-exome sequencing (WES), and medical geneticists may recommend them after consulting with families in prenatal cases [1].

A prenatal whole-exome sequencing test examines approximately 1–2% of the fetal genome and can achieve a diagnostic yield ranging from 6.2% to 80%, depending on the selected cohort [2–5]. Prenatal exome testing implementation varies considerably across countries and continents, with discrepancies even within the same continent in turnaround time, cost management, test indications, and result interpretation.

Current practice guidelines highlight the importance of using molecular karyotyping or chromosomal microarray analysis (CMA) as gold-standard, first-tier tests for pregnancies with ultrasound-detected malformations [6,7]. This technique has a diagnostic yield of only 30–40% based on international studies [8–11].

In 2018, the International Society for Prenatal Diagnosis (ISPD), the Society for Maternal-Fetal Medicine (SMFM), and the Perinatal Quality Foundation (PQF) presented a joint position statement regarding the use of genome-wide sequencing for fetal diagnosis [12]. The societies highlighted the importance of next-generation sequencing (NGS) after chromosomal microarray analysis (CMA) has been performed and yielded an uninformative result, or its concurrent use in specific cases, while stating that routine sequencing is not sustainable and should not be considered a first-tier test for all genetic conditions [12].

The objective of the present study was to assess the clinical utility of prenatal whole-exome sequencing in selected patients within Romanian prenatal care, with a particular focus on its diagnostic effectiveness compared with molecular karyotyping, especially in patients with adverse pregnancy outcomes and negative results from standard genetic testing, such as molecular karyotyping. The findings may contribute to the existing literature and support Romanian national authorities in considering WES testing for selected cases, as is currently offered in several countries, including England through the National Health Service (NHS), Israel, the Netherlands, France in academic hospitals, Germany, Belgium, and North America. Currently, prenatal WES is not routinely reimbursed in Eastern European countries such as Romania, limiting access to this testing in many cases.

The present study also aimed to compare the diagnostic yield observed in the Romanian cohort with those reported internationally for prenatal WES [13–17].

## Material and Methods

We present a retrospective analysis of indications, genetic testing results (including molecular karyotyping and prenatal WES), and the impact of molecular findings on couples and the multidisciplinary teams involved in their care.

The inclusion criteria were: (1) patients of Romanian Caucasian descent; (2) a prenatal sample obtained through an invasive procedure, either amniocentesis or chorionic villus sampling, or from a product of conception; (3) the fetus presenting with at least one major or two or more structural malformations visible on ultrasound; and (4) testing performed at Synevo Romania between January 2024 and March 2026, representing consecutive cases. The team offered the option to report carrier variants or secondary findings, in accordance with the 2023 recommendations (version 3.2) of the American College of Medical Genetics and Genomics (ACMG) for reporting secondary findings, only when families provided consent and opted in [18]. From September 2025, the team incorporated the updated ACMG recommendations (version 3.3) for reporting secondary findings [19]. Ten consecutive referred cases met the inclusion criteria. None underwent single-nucleotide polymorphism (SNP)-array testing alone; however, SNP-array analysis was performed before WES when clinically indicated.

After selecting pregnancies with abnormal ultrasound findings not suggestive of a well-established genetic syndrome, the testing algorithm used molecular karyotyping as the initial test when clinically indicated. In cases with a negative molecular karyotyping result, prenatal WES was subsequently performed.

Genomic DNA was extracted using the QIAamp DNA Mini Kit (Qiagen) according to the manufacturer’s protocol. DNA concentration was measured using Qubit fluorometry, and DNA purity was assessed using NanoDrop spectrophotometry. WES Mono (fetus) or Trio (fetus and parents) was performed by massively parallel sequencing of the whole coding region on an Illumina NovaSeq X platform (average coverage >100×; read length 2 × 100 bp). The Twist Human Core Exome kit with the RefSeq and mitochondrial panel (Twist Bioscience, San Francisco, CA, USA) was used for library preparation. Bioinformatic analysis compared the test sample with the human reference sequence (hg38) using Illumina’s DRAGEN Bio-IT and Franklin (Qiagen), together with the OMIM, ClinVar, gnomAD, LOVD, and HGMD databases. Variants with a minor allele frequency <0.01 were prioritized, and interpretation incorporated phenotype-driven filtering using Human Phenotype Ontology (HPO) terms. To exclude maternal cell contamination, maternal peripheral blood was tested in parallel. Over 99% of target bases were covered at ≥20× and 97% at ≥30×; the analytical sensitivity for single-nucleotide variants (SNVs) exceeded 99% for regions covered at ≥20× on internal validation. Copy-number variants (CNVs) were assessed from WES data and none were found. As the accredited laboratory had validated these NGS workflows, diagnostic variants were considered high-confidence and did not require routine orthogonal confirmation. Variants were classified according to the ACMG/AMP 2015 guidelines, independently reviewed by two clinical geneticists, and curated by a team of bioinformaticians before interpretation. Testing was performed in an ISO 15189-accredited laboratory that participates in external quality-assessment programs such as GenQA.

The turnaround time was under 3 weeks. When the result was ready, the family was scheduled for a medical genetics consultation to understand it and make an informed decision about managing the pregnancy. All families declared no consanguinity. Raw sequencing data have been deposited in a public repository – ClinVar, under submission number SUB16200327.

We obtained informed consent from every family after a medical genetics consultation, once we explained the methodology, capabilities, and limitations of the test. Pre-test counseling covered the right not to know, possible adult-onset genetic disorders, and the option to report secondary findings; families could also opt in to receive carrier status, to inform the management of future pregnancies and the testing of at-risk relatives. After the results, a post-test genetic counseling session was held, at which the results were explained, and a written report with recommendations was prepared for the multidisciplinary team caring for the family.

## Results

**Figure 1:**
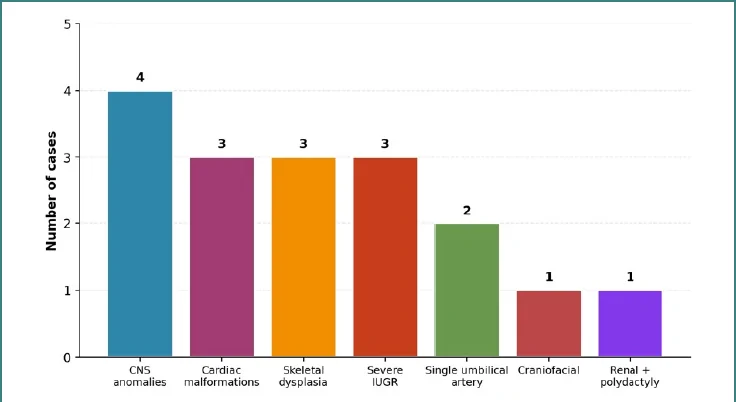
Distribution of fetal structural anomalies among the ten pregnancies included in the study

**Figure 2:**
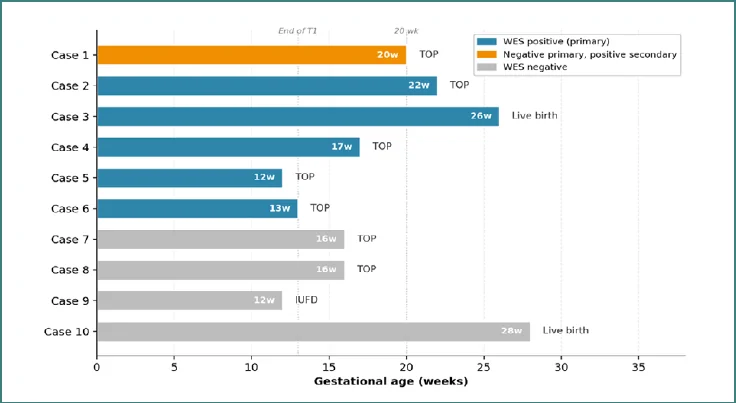
Gestational age at testing and WES outcome for each pregnancy

Ten cases tested by prenatal WES were included after meeting the criteria above. Microarray was first performed in 8 of the 10 cases with suspected copy number variations and yielded no findings, so the team proceeded with prenatal exome sequencing. The results are shown in Table 1 and Figures 1 and 2.

**Table 1:** Clinical and molecular findings in the ten pregnancies that underwent prenatal whole-exome sequencing

Case	Fetal phenotype	GA	Previous tests	Fetal WES result	Pregnancy outcome	Incidental / Secondary Findings/carrier status
1	Dandy-Walker malformation triad: hydrocephalus, posterior fossa cyst and cerebellar vermis dysgenesis	20 weeks	Negative SNP array	Negative primary finding Positive secondary variant WES MONO	Medical termination of pregnancy	Secondary finding: P variant *MYBPC3*: c.26-2A>G p.? - Het (AD)
2	Single umbilical artery, Cardiac complex malformation: apex is formed by right ventricle, hypoplastic left heart, fetal posterior fossa malformation: communication between the fourth ventricle and the cisterna magna	22 weeks	Negative SNP array	*P* variant *KMT2D* : c.16342C>T p.(Arg5448*) - Het (AD) WES MONO	Medical termination of pregnancy	Secondary findings: *P* variant *HFE*:c.187C>G p.His63Asp - Homozygous (AR)
3	Asymmetric shortening of the long bones <1^st^ percentile	26 weeks	—	*P* variant *COL2A1* : c.3469_3486dup p.(Ala1157_Pro1162dup) - Het (AD) WES MONO	Baby born with asymmetric shortening of long bones; apparently with no other complications and monitored by the pediatrics team	—
4	Fetal skeletal dysplasia involving both upper and lower limbs	17 weeks	—	*P* variant *COL1A2* :c.1971+1G>C p.? - Het (AD) WES MONO	Medical termination of pregnancy	Incidental findings: *ADCY6* : c.2996A>G p.(Asn999Ser) — Het (AR) ADCY6 : c.85C>T p.(Arg29Trp) — Het (AR)
5	Encephalocele, polycystic kidneys, and polydactyly, suspicion of Meckel–Gruber syndrome.	12 weeks	Negative SNP array	*P* variant *CC2D2A*:c.4675-1G>C p.? Homozygous (AR) WES MONO	Medical termination of pregnancy	Carrier status: *P* variant *HJV* : c.959G>T p.(Gly320Val) - Het (AR)
6	Absent nasal bone, long philtrum, cleft palate, and intrauterine growth restriction	13 weeks	Negative SNP array	*P* variant *NBAS*:c.[539_540delinsTG;619C>T] p.[Ser180Met;Arg207*] - Het (AR) “hot VUS” variant NBAS: NC_00002.12 (NM_15909.4):c.6573-1047A>G p.? – Het (AR) WES MONO	Medical termination of pregnancy	Incidental finding: *P* variant *PROKR2* : c.58del (p.His20Metfs*24) - Het (AD)
7	Severe intrauterine growth restriction and cardiac malformation: hypoplastic left heart	16 weeks	Negative SNP array	Negative WES MONO	Medical termination of pregnancy	—
8	Complex cardiac malformation: ventricular septal defect, Mitral and Aortic valve atresia, double outlet right ventricle	16 weeks	Negative SNP array	Negative WES MONO	Medical termination of pregnancy	—
9	Nuchal translucency=7.5, single umbilical artery	12 weeks	Negative SNP array	Negative WES TRIO	Pregnancy stopped in evolution	—
10	Severe asymmetric IUGR, with long bones < 1^st^ percentile, hypoplasia of corpus callosum	28 weeks	Negative SNP array	Negative WES MONO	Born, affirmatively healthy, further follow-up needed	—

AD, autosomal dominant; AR, autosomal recessive; GA, gestational age; Het, heterozygous; IUGR, intrauterine growth restriction; P, pathogenic; SNP, single-nucleotide polymorphism; VUS, variant of uncertain significance; WES, whole-exome sequencing (MONO, fetus only; TRIO, fetus and both parents).

The series comprised four cases with central nervous system (CNS) anomalies, three with cardiac malformations, three with severe intrauterine growth restriction, three with skeletal dysplasias, and two with a single umbilical artery. Of the 8 cases with a prior negative SNP array, WES identified a pathogenic variant in four (50% of those previously tested by CMA), and three of these four were primary findings that could explain the phenotype (37.5%). This supports the 2018 position statement of the International Society for Prenatal Diagnosis, the Society for Maternal–Fetal Medicine, and the Perinatal Quality Foundation, which recommends exome sequencing after a non-informative CMA in cases with an ultrasound anomaly. Importantly, the two cases in which WES was used as a first-tier test—Cases 3 and 4, both with suspected skeletal dysplasia and a suggestive phenotype—illustrate that, for certain highly suggestive phenotypes, a direct monogenic approach is both diagnostically and logistically more efficient, consistent with current international recommendations.

We considered a case positive when WES identified a primary finding that could explain the phenotype (pathogenic or likely pathogenic); we also counted as positive the case with a second “hot” variant of uncertain significance (VUS) completing a recessive genotype (Case 6).

The molecular diagnostic yield was positive for five cases, as primary findings overall, meaning a percentage of 50%, with four cases displaying a Mendelian autosomal dominant disorder and one case showing an autosomal recessive disorder. The yield after a negative SNP array was 37.5% for primary findings, and the yield when WES was used as a first-line test for a highly suggestive monogenic phenotype was 100% (two of two cases; cases 3 and 4).

Among our patients, two cases had secondary findings (20%), in one of which we found a homozygous variant in the *HFE* gene, not the one present in the SF V3.3 ACMG list, as seen in Case 2. Two other fetuses were positive for incidental findings (Cases 4 and 6), representing 20%. Although we followed ACMG guidelines, in cases where the parents decided termination of pregnancy, after this decision, the team agreed to report carrier status of the fetuses for screening purposes and for cascade testing of relatives. One fetus was a carrier of an *HJV* variant (Case 5).

The clinical impact of the prenatal WES test was medical termination of pregnancy (TOP), in Cases 1,2,4,5 and 6 (50% of cases). In two further cases (7 and 8), TOP was conducted based on ultrasound manifestations, as WES yielded negative results. One pregnancy stopped in evolution, and WES couldn’t explain the phenotype; in another case in which WES was negative, the baby was apparently born healthy, so further follow-up is needed. In one positive case (Case 3), the family chose to continue the pregnancy, but WES was very helpful in diagnosis, counseling, and preparing the multidisciplinary team for delivery and pregnancy management.

Other important aspects of the clinical impact were genetic counseling and recurrence-risk estimation, based on the known mode of inheritance of each disorder. The team recommended cascade testing of all first-degree relatives in every positive case; at the close of the study, none had undergone segregation analysis except Case 6, whose parents had each been shown previously to carry one of the *NBAS* variants identified in the fetus.

**Figure 3:**
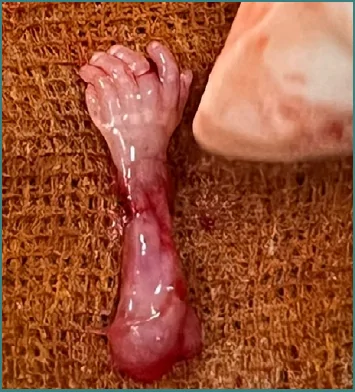
Case 5 — postaxial polydactyly in a fetus with suspected Meckel–Gruber syndrome, confirmed on pathological examination. Published with parental consent.

Some products of conception were sent for pathological examination, which confirmed the ultrasound findings — for example, the postaxial polydactyly in Case 5 (Figure 3).

## Discussion

Managing secondary and incidental findings remains a key component of prenatal genomic testing, with major consequences for informed decision-making and genetic counseling. In our group of patients, thorough pre-test counseling empowered all families to make informed decisions about the disclosure of secondary and incidental findings, and all participants chose to receive these results. Moreover, all families opted to obtain carrier status information after childbirth or medical termination of pregnancy, highlighting the recognized importance of extensive genomic data beyond the initial diagnostic purpose.

Our referral pathway, involving gynecologists, obstetricians, medical geneticists, and maternal-fetal medicine specialists, highlights the multidisciplinary nature of prenatal genomic diagnostics and the importance of coordinated clinical assessment to facilitate timely access to testing.

A significant challenge in prenatal genomic medicine is the turnaround time (TAT) needed to produce and analyze sequencing outcomes. In alignment with global trends, TAT has steadily improved because of advancements in sequencing technologies, refined laboratory processes, growing proficiency in variant analysis, and the incorporation of AI-driven bioinformatics tools. Nonetheless, further reductions in TAT are crucial in the prenatal context because the therapeutic relevance of genetic testing is inherently connected to the gestational age at diagnosis.

This is especially important when fetal structural anomalies are first detected in the second trimester, particularly after 20 weeks of gestation. In numerous European jurisdictions, reproductive options may be significantly restricted by legal restrictions on termination of pregnancy after this gestational age, unless the fetal condition is significantly associated with maternal or neonatal risk. Delays in obtaining a molecular diagnosis may directly influence the range of management options available to prospective parents, as many monogenic disorders exhibit considerable phenotypic variability in the prenatal period and are associated with significant long-term morbidity rather than immediate fetal or maternal compromise. These results underscore the need for early identification of fetal anomalies, prompt referral for genomic evaluation, and rapid access to high-quality prenatal sequencing to optimize the clinical utility of genomic diagnostics and support informed reproductive decision-making. [20]

Limitations include the small sample size (though important for a country like Romania), the retrospective design, incomplete cascade testing of parents and other first-degree relatives, incomplete postnatal follow-up, and the fact that all data were collected from a single reference laboratory. Given the descriptive nature of this study and the small sample size, we did not perform inferential statistical analyses.

## Conclusion

In conclusion, prenatal whole-exome sequencing yielded a 50% diagnostic yield in our case series, underscoring its clinical significance for fetuses with structural abnormalities. This diagnostic efficacy aligns with previously published global data and our prior experiences in Romania, reinforcing the consistency of this method across various clinical environments. In addition to providing a molecular diagnosis, prenatal whole-exome sequencing offers clinically relevant insights that enhance genetic counseling, improve recurrence risk evaluation, and support collaborative decision-making about the current pregnancy and future reproductive strategies.

Notably, a negative whole-exome sequencing result is not incompatible with an underlying genetic etiology. Some patients may benefit from additional genomic investigations, including epigenetic, transcriptomic, or other advanced molecular analyses, when guided by the clinical phenotype, therefore improving diagnostic precision.

Despite variability in the diagnostic yield of prenatal genomic testing influenced by factors such as fetal phenotype, affected organ systems, family history, and case selection, our results contribute to the growing evidence supporting the incorporation of whole-exome sequencing into the diagnostic process for carefully selected pregnancies with fetal structural anomalies. Future multicenter investigations involving larger and more phenotypically varied groups are needed to more accurately delineate the clinical contexts in which prenatal whole-exome sequencing offers the greatest diagnostic and clinical advantages, and to strengthen evidence-based guidelines for its integration into standard prenatal care. The diagnostic yield observed in this cohort may partly reflect referral bias, as only highly selected pregnancies meeting predefined criteria underwent prenatal WES. These preliminary findings support further prospective multicenter studies evaluating prenatal WES in Romanian clinical practice.

## Data Availability

The datasets generated and/or analyzed during the current study are available in the ClinVar repository, accession number SCV007596795-SCV007596806.

## Personal thanks

We acknowledge the families of the patients for their support.
